# 1140. Impact of an Implementation Science Initiative to Improve Vaccine Confidence for Invasive Meningococcal Disease (IMD) Prevention within American Indian (AI) Communities

**DOI:** 10.1093/ofid/ofad500.981

**Published:** 2023-11-27

**Authors:** Lyle Ignace, Ruth Hursman, Jenniffer A Meza Jimenez, Jeffrey D Carter, Abigail K Corona, Bonnie Douglas, Laura Simone, Leah Molloy

**Affiliations:** Gerald L. Ignace Indian Health Center, Milwaukee, Wisconsin; EXCITE project - Extension Foundation, BISMARCK, North Dakota; PRIME Education, West Palm Beach, Florida; PRIME Education, LLC, Fort Lauderdale, Florida; PRIME Education, LLC, Fort Lauderdale, Florida; PRIME Education, LLC, Fort Lauderdale, Florida; PRIME Education, LLC, Fort Lauderdale, Florida; PRIME Education, LLC, Fort Lauderdale, Florida

## Abstract

**Background:**

Meningococcal vaccine uptake among adolescents remains suboptimal. This project aimed to identify barriers and motivators for vaccination in AI communities.

**Methods:**

Between 12/2022 and 2/2023, 3 live family education sessions were held within AI communities in WI and ND. Healthcare provider (HCP) participants also attended a workshop to review current vaccine recommendations. Surveys were administered before/after the workshop and family education sessions.

**Results:**

Surveys were completed by 56 family members. Baseline confidence in vaccine safety, efficacy and benefit was low, which increased after the sessions (Fig. 1). Parent understanding that the vaccine cannot cause disease improved from 51% to 82% (p =.001). Top reported barriers to vaccination were concern for immediate side effects and long-term side effects, and low perceived risk for IMD (34%, 26%, 25%, respectively). Parents identified learning more about IMD (40%), friends/family getting vaccinated (19%), and a local outbreak (12%) as top motivators to seek vaccination. When asked what they thought would motivate families to seek vaccination, HCPs leading the sessions (n=11) identified learning more about IMD (82%) and friends/family getting vaccinated (27%) only. HCP confidence in counseling families was low and improved after the sessions (Fig. 2). HCPs attending the workshop (n=28) reported discomfort contradicting patients/families as the top challenge in addressing vaccine hesitancy, and more HCPs felt confident having conversations to reduce vaccine hesitancy after the workshop (80%) than before (28%). HCP knowledge improved after the workshop, and more HCPs understood that MenACWY booster doses are not needed when initial vaccination occurs at age ≥16 (50%), and correctly identified “provider recommendation” as the top literature-reported factor influencing parental vaccine decisions (50%) compared to baseline (32% and 14%, respectively). HCP action plans for after the sessions prioritized routinely discussing vaccines at well visits (80%), providing written materials (50%) and staying current on new vaccines (50%).

**Figure:**
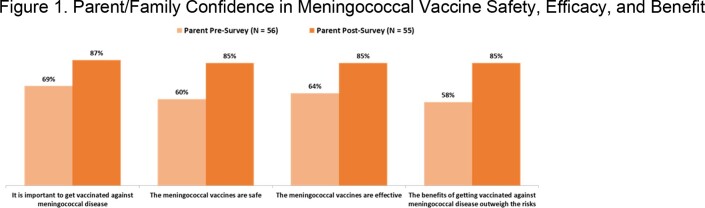

**Figure:**
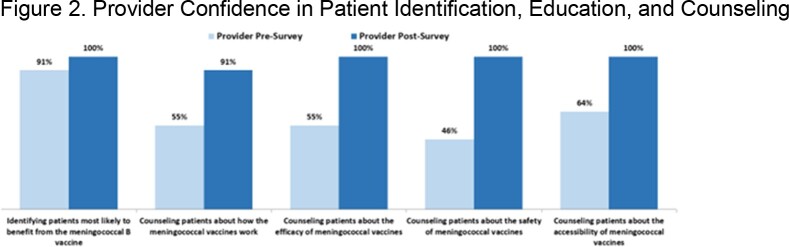

**Conclusion:**

Parent and provider knowledge and confidence improved after the program and offered insights into community-level motivators and barriers to vaccination.

**Disclosures:**

**All Authors**: No reported disclosures

